# Effects of Dietary Cobalt Levels on Growth Performance, Antioxidant Capacity, and Immune Status of Juvenile Largemouth Bass (*Micropterus salmoides*)

**DOI:** 10.3390/vetsci11110576

**Published:** 2024-11-18

**Authors:** Dongyu Huang, Joshua Daniel Jahazi, Mingchun Ren, Lu Zhang, Hualiang Liang

**Affiliations:** 1 Key Laboratory of Integrated Rice-Fish Farming Ecology, Ministry of Agriculture and Rural Affairs, Freshwater Fisheries Research Center, Chinese Academy of Fishery Sciences, Wuxi 214081, China; huangdongyu@ffrc.cn (D.H.);; 2 Wuxi Fisheries College, Nanjing Agricultural University, Wuxi 214081, China; 3 Tongwei Agricultural Development Co., Ltd., Key Laboratory of Nutrition and Healthy Culture of Aquatic Livestock and Poultry, Ministry of Agriculture and Rural Affairs, Healthy Aquaculture Key Laboratory of Sichuan Province, Chengdu 610093, China

**Keywords:** cobalt, growth performance, antioxidant capacity, immune status, largemouth bass

## Abstract

A 9-week experiment investigated the effects of dietary cobalt levels on the growth performance, antioxidant capacity, and immunity of largemouth bass. Six feed groups were designed and each group received different cobalt levels, including 0.129 mg/kg (control group), 0.192 mg/kg, 0.201 mg/kg, 0.233 mg/kg, 0.277 mg/kg, and 0.316 mg/kg. The results show that the control group (0.129 mg/kg diet) had the lowest final body weight (FBW), weight gain rate (WGR), and specific growth rate (SGR), and the highest feed conversion ratio (FCR), when compared to the cobalt supplementation groups. Dietary cobalt levels of 0.192 mg/kg increased the body protein content and decreased the body moisture content. Regarding antioxidant capacity, the highest catalase (CAT) activity was found in the 0.277 mg/kg dietary cobalt group, while the malondialdehyde (MDA) content was significantly diminished; the total antioxidant capacity (T-AOC) content and glutathione peroxidase (GSH-Px) activity exhibited the highest values in the 0.192 mg/kg and 0.233 mg/kg dietary cobalt groups, respectively. Regarding gene expression, compared with the control group, the mRNA expression of *sod* was upregulated in the 0.192 mg/kg, 0.233 mg/kg, and 0.277 mg/kg dietary cobalt groups, while the mRNA expression of *gpx* was diminished when dietary cobalt levels were below 0.233 mg/kg. In addition, the highest *il-10* and *tgf-β* mRNA expression levels were observed in the 0.201 mg/kg and 0.233 mg/kg dietary cobalt groups, respectively. According to the quadratic regression analysis based on the SGR and FCR, the optimal requirement was 0.24 and 0.26 mg/kg of dietary cobalt, respectively.

## Simple Summary

A 9-week experiment aimed to investigate the effects of dietary cobalt levels on the growth performance, antioxidant capacity, and immunity of largemouth bass. According to the results of growth performance, whole-body composition, antioxidant parameters in the liver, gene expression of the Nrf2 signaling pathway, and NF-κB signaling pathway, it was found that there was an improvement in the growth performance and feed utilization of largemouth bass fed with optimal dietary cobalt levels. Furthermore, cobalt has an important function in improving the antioxidant status and immune response of largemouth bass. Quadratic regression analyses based on the SGR and FCR showed that the optimal requirement was 0.24 and 0.26 mg/kg of dietary cobalt, respectively.

## 1. Introduction

Largemouth bass (*Micropterus salmoides*), a member of the genus *Micropterus* in the sunfish family centrachidae of perciformes, is native to the Mississippi River system in California, United States [1]. It reaches sexual maturity at more than one year of age (around 131 g body weight) and reproduces once per year [2]. Largemouth bass was first introduced in mainland China in Guangdong province in 1980 [3], and its production has expanded dramatically, reaching more than 880,000 tons by the year 2023 [4].

As in other fish species, largemouth bass needs minerals (phosphorus, selenium, etc.) for different physiological activities and biochemical functions [5,6]. However, there is limited research thus far on the dietary cobalt requirements of aquatic species. Cobalt is an important component of cyanocobalamin (vitamin B12), which is implicated in nitrogen assimilation and the synthesis of muscle proteins, and has growth-promoting properties [7]. Furthermore, cobalt, as a trace mineral, is required by most animals for vitamin synthesis through gut microflora and bacteria [8,9], and it is commonly used as a feed additive in animal nutrition [10]. In the current study, cobalt chloride and cobalt nitrate were mainly used as cobalt sources to investigate the cobalt requirements of fish. In common carp (*Cyprinus carpio*), cobalt chloride is said to improve the growth rate and significantly increase the crude protein content when up to 1.5% is added to a supplementary diet [11,12]. In silver sea perch (*Lates calcarifer*) and Asian catfish (*Clarias batrachus*), a 2.5 mg/kg cobalt and 45 mg/kg zinc diet could improve growth performance [13]. Additionally, in juvenile white shrimp (*Litopenaeus vannamei*), a 10 mg/kg dietary supplementation of cobalt methionine enhanced growth performance [14]. However, the dietary cobalt requirement for largemouth bass has not yet been studied, and there is an urgent need to improve this database.

Previous studies have explored the roles of minerals on antioxidant capacity and immunity improvement in aquatic animal species. Other microminerals, such as selenium, are reportedly advantageous in aquaculture when used in an acceptable optimal range; selenium is vital for growth performance and immunity enhancement in certain fish, with varying dietary acceptability [15]. Similarly to the results of previous research, a study demonstrated that dietary cobalt yielded significantly higher values in antioxidant enzymes when added to the diet of Nile tilapia (*Oreochromis niloticus*) [16]. A previous study showed that appropriate levels of Co supplementation could improve the immune capacity of Golden mahseer (*Tor putitora*), whereas excessive Co additions could negatively affect the immune response, probably due to oxidative stress and the production of pro-inflammatory factors [17]. It is therefore of interest whether different dietary cobalt levels affect the antioxidant capacity and immune response of largemouth bass.

The main purpose of this study was, thus, to determine the optimal cobalt requirement for juvenile largemouth bass, and assess the effect of dietary cobalt levels on growth performance, antioxidant capacity, and immune status.

## 2. Materials and Methods

### 2.1. Experimental Diet

Six feed groups were formulated at the Freshwater Fisheries Research Center (FFRC). Six cobalt addition levels of 0, 0.1, 0.2, 0.3, 0.4, and 0.5 mg/kg in the diets were prepared with reference to the feed formula of largemouth bass reported by Song et al. [18] by adding cobalt chloride to the base formulation (Table 1). Through measurement and analysis, the following concentrations of dietary cobalt were determined: 0.129 (control group), 0.192, 0.201, 0.233, 0.277, and 0.316 mg/kg, respectively. All six isonitrogenous and iso-lipidic diets were weighed according to the formulation table, the ingredients were mixed, and water was added; the mix was used to prepare 1.5 mm caliber pellets using a pelleting machine (F-26(II); South China University of Science and Technology, Guangzhou, China), and finally, the feeds were ventilated, dried, and bagged in a cool place outdoors, and stored at −20 °C until their subsequent use.

### 2.2. Feeding Management

The feeding practice system used was a series of recirculation aquaculture system (RAS) tanks at FFRC (Yixing base). Six treatments were set, each having triplicate samples. Each treatment used 3 tanks, comprising a total of 18 tanks with a volume of 270 L each. Juvenile largemouth bass were provided by Chia Tai Aquatic Products Co. Ltd. (Huzhou, China). Twenty fish (average weight: 1.67 ± 0.03 g) were stocked in each tank. The fish were acclimatized for seven days and thereafter fed twice a day for nine weeks to achieve significant satiety. During the feeding trial, the water quality indicators were detected by the Octadem Multi-Parameter Water Quality Analyser (Type OCT-A) (Wuxi Octadem Biotechnology Co., LTD, Wuxi, China) daily, the pH ranged from 7.5 to 8.0, the dissolved oxygen content was larger than 6.0 mg/L, and the water temperature was maintained at 30 ± 2 °C.

### 2.3. Sample Collection

After a 9-week feed, the fish were starved for 24 h before sampling. All fish from each tank were taken to record the total number and weight of the fish. Then eight fish were randomly selected, and liver samples were taken from three of the fish and stored at −80 °C before antioxidant parameters and qPCR analysis. In addition, another five fish per tank were stored at −20 °C for the whole-body proximate analysis.

### 2.4. Proximate Chemical Composition Analyses

The approximate dietary composition and body composition were determined according to the AOAC method [19]. The moisture determination was carried out by oven drying at 105 °C. The crude lipid content was determined by using Soxhlet extraction, and the crude protein content was analyzed via the Kjeldahl method. The ash content was obtained using a muffle furnace at 550 °C.

### 2.5. Liver Antioxidant Parameters

Catalase (CAT), superoxide dismutase (SOD), glutathione (GSH), glutathione peroxidase (GSH-Px), total antioxidant capacity (T-AOC), and malondialdehyde (MDA) were analyzed by using assay kits from Jian Cheng Bioengineering Institute, Nanjing [20]. The CAT activity was measured by the ammonium molybdenum acid method, the activity of SOD was determined with the WST-1 method, the microplate method was used for the determination of GSH, the GSH-Px activity was measured by the colorimetric method, the T-AOC was determined by the ABTS method, and the MDA content was measured by the TBA method.

### 2.6. qPCR Analysis

RNA extraction of liver tissues was performed using the RNAiso Plus reagent (Vazyme Biotech Co., Nanning, China), and its concentration was determined using a Nanodrop 2000 spectrophotometer (Waltham, MA, USA). The concentration of the RNA samples was adjusted to 60 ng/µL, with A260/A280 ranging between 1.8 and 2.0. All gene expressions were determined using an SYBR Green Kit (Vazyme Biotech Co., Nanning, China), and quantification was performed using a PCR machine (CFX96 Real time system, Bio-Rad, Singapore). *gapdh* was used as the reference gene; it has previously been used in a largemouth bass study [21], and no significant changes were found in this study or the previous study. Table 2 presents the primer sequences for the genes; the relative standard curve method was used to quantify mRNA expression.

### 2.7. Statistical Analysis

All data were subjected to normality and homogeneity tests using Levene’s test where necessary, and then the experimental data (means ± SE) were analyzed using SPSS v. 20.0 (IBM Corp., Armonk, NY, USA) statistical software for one-way analysis of variance (ANOVA). Mean testing within the groups was performed using Duncan’s test with a *p* < 0.05 significance level, and the results were reported as mean ± standard error (SE) values. The quadratic regression model was selected to determine the optimum dietary cobalt requirement by comparing the estimation coefficient (R^2^) among the linear regression model (SGR, 0.641; FCR, 0.457), quadratic regression model (SGR, 0.952; FCR, 0.957), and broken-linear regression model (SGR, 0.911; FCR, 0.956).

## 3. Results

### 3.1. Growth Performance and Feed Utilization

**Figure 1:**
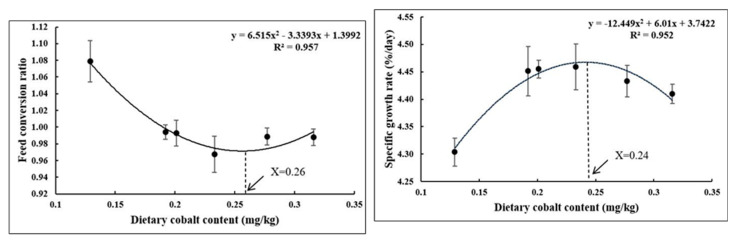
Optimal cobalt requirement of juvenile largemouth bass.

Table 3 shows that the weight gain rate (WGR) and specific growth rate (SGR) were affected by dietary cobalt levels. The control group (0.129 mg/kg diet) showed significantly lower WGR and SGR than all other groups (*p* < 0.05), unlike the feed conversion ratio (FCR), where it showed significantly higher values than all other groups (*p* < 0.05). In addition, dietary cobalt levels did not affect the survival rate (SR) within groups (*p* > 0.05). Quadratic regression analyses based on SGR (R^2^ = 0.952) and FCR (R^2^ = 0.957) showed that the optimal dietary cobalt requirement was 0.24 and 0.26 mg/kg of diet, respectively (Figure 1).

### 3.2. Whole-Body Composition

Table 4 shows that dietary cobalt levels did not have effects on the lipid and ash contents of the whole body (*p* > 0.05). The crude protein content of the whole body showed a higher value when the dietary cobalt level was 0.192 mg/kg as opposed to moisture content values, which were significantly higher than the control group (*p* < 0.05).

### 3.3. Antioxidant Indicators in Liver

The results in Table 5 show that when the dietary cobalt level was 0.192 mg/kg, the level of T-AOC was significantly higher than that in other groups (*p* < 0.05), and the level of CAT in the 0.277 mg/kg dietary cobalt level group was significantly higher than that in the control group (*p* < 0.05). When the dietary cobalt level was 0.233 mg/kg, the level of GSH-Px was significantly higher than that in the control group (*p* < 0.05), and the MDA content in the 0.277 mg/kg and 0.316 mg/kg dietary cobalt level groups was significantly lower than that in the 0.201 mg/kg dietary cobalt level group (*p* < 0.05). The levels of SOD and GSH were not affected by dietary cobalt levels (*p* > 0.05).

### 3.4. Gene Expression of Nrf2 Signaling Pathway

**Figure 2:**
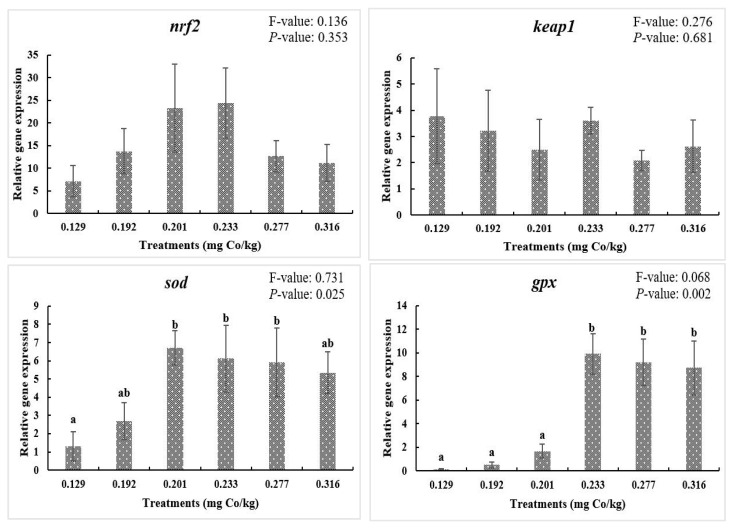
Relative gene expressions of Nrf2 signaling pathway. F-value, Homogeneity of Variance Test. Results are shown as mean values and standard error (±SE) (*n* = 9), significant differences between the six treatments are denoted by different letters (*p* < 0.05).

Figure 2 shows that dietary cobalt levels did not affect the nrf2 and keap1 mRNA expression levels (*p* > 0.05). In addition, the *sod* mRNA levels were markedly upregulated at the 0.201 mg/kg, 0.233 mg/kg, and 0.277 mg/kg dietary cobalt levels compared with the control group (*p* < 0.05). Interestingly, this phenomenon was apparent when the dietary cobalt level reached 0.233 mg/kg, and the mRNA levels of *gpx* were upregulated compared with the control group (*p* < 0.05).

### 3.5. Gene Expression of NF-κB Signaling Pathway

**Figure 3:**
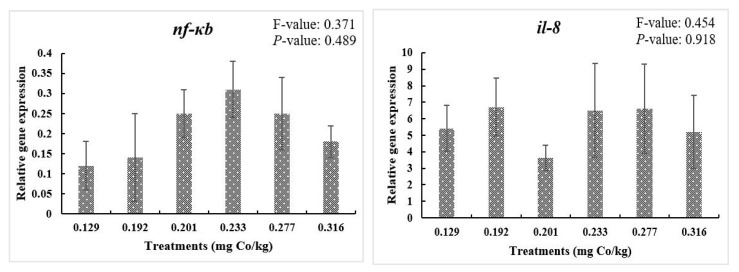

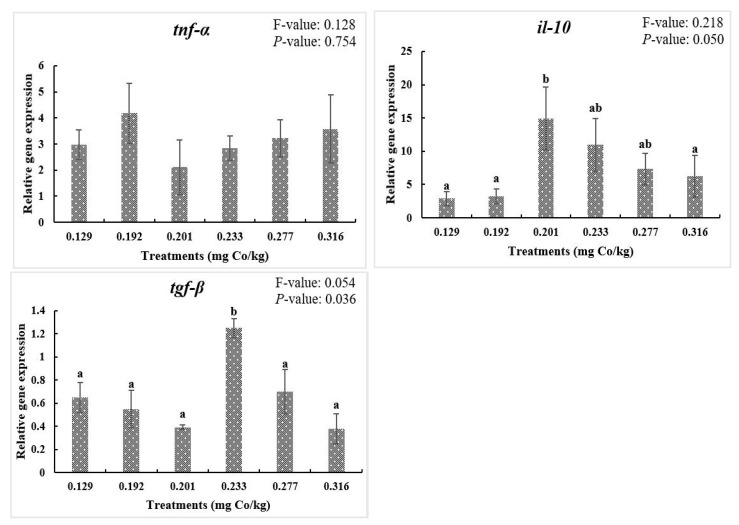
Relative gene expressions of NF-κB signaling pathway. F-value, Homogeneity of Variance Test. Results are shown as mean values and standard error (±SE) (*n* = 9), significant differences between the six treatments are denoted by different letters (*p* < 0.05).

As shown in Figure 3, dietary cobalt levels did not affect the *nf-kb*, *il-8*, and *tnf-α* mRNA expression levels (*p* > 0.05). Moreover, the mRNA levels of *il-10* at the 0.201 mg/kg dietary cobalt level exhibited a higher value than the control group (*p* < 0.05). In addition, the mRNA levels of *tgf-β* were also higher at the 0.233 mg/kg dietary cobalt level than all the remaining levels (*p* < 0.05).

## 4. Discussion

In aquatic organisms, specifically fish species, it has been reported that there is a positive correlation between the mineral content in a diet and growth performance parameters. Although studies on trace minerals are limited, research in this field is ongoing. Previous studies on the importance of trace minerals in aquaculture nutrition have shown that dietary cobalt has an impact on growth in carp and rainbow trout as well as an improvement in survival rates in gold spot mullet (*Liza parsia*) [24]. Furthermore, in Golden mahseer, the addition of 3 mg/kg dietary cobalt significantly affected the growth performance with a 37.52% weight gain compared to the control group [9]. In our study, dietary cobalt levels (0.192 mg/kg–0.316 mg/kg) were shown to improve the growth parameters and reduce the FCR as compared to the control group (0.129 mg/kg diet). Furthermore, it was recommended that the supplementation of cobalt should not exceed 1 mg/kg of complete feed for all animal species except fish [10]. The range for the optimal cobalt requirement for different fish species is 0.05–5 mg/kg diet [25]. In this study, the requirement of cobalt for largemouth bass is 0.24–0.26 mg/kg diet based on FCR and SGR. These results fully demonstrate the importance of optimal dietary cobalt levels in promoting fish growth.

Furthermore, it was shown that the body protein content increased and the moisture content decreased at 0.192 mg/kg cobalt in the diet compared to the control. In this study, the increased protein content in fish body composition was assisted by the supplementation of dietary cobalt, which facilitates amino acid incorporation into the fish body [9]. Similarly, another study showed an increase in crude protein content in carp fed cobalt supplements [26]. In addition, dietary cobalt levels did not affect the composition of crude ash and crude lipid contents in this study. These results were slightly perplexing as we expected to observe an increased ash content due to the addition of cobalt as a trace mineral. It has also been found that the level of the mineral magnesium does not make a difference in body ash content [27], which is **similar to** our findings. The probable reason for these results is that under specific conditions of appropriate mineral content and balanced ratios in the feed, fish can effectively absorb these minerals without affecting the ash content of the fish. In addition, there are many reasons for differences in body composition, and differences in breeding environment and individual size are all potential influencing factors.

Biologically, antioxidants are compounds or substances that inhibit damage to body cells by free radicals as a result of oxidation [28]. Antioxidant substances actively fight against free radicals to prevent cell damage, and they are named antioxidants due to their ability to break down the process of oxidation [29,30]. In previous studies, minerals such as selenium, zinc, manganese, and iron have been found to play an important role in enzymatic reactions [31], have antioxidant effects, and can help reduce free radical damage and cellular oxidative stress [32,33,34,35]. In our study, appropriate levels of cobalt were shown to improve antioxidant capacity, which is supported by the results of relevant antioxidant indicators. As reported, cobalt is an essential trace element for organisms and is associated with a variety of enzyme activities [9]. In our experiment, higher CAT and GSH-Px activities were observed at 0.277 mg/kg and 0.233 mg/kg dietary cobalt levels, respectively. In addition, the T-AOC content showed a significant increase when the dietary cobalt level was 0.192 mg/kg, which was considerably more significant than the control group. Similar results for some parameters were obtained in previous studies, whereby cobalt exposure in Japanese flounder (*Paralichthys olivaceus*) resulted in significantly higher CAT activity when the cobalt level was 5 mg/L than that in the control group [36]. Liu et al. [37] reported that appropriate dietary cobalt levels could significantly increase the hepatic T-AOC content and GSH-PX activity in pearl gentian grouper (*Epinephelus lanceolatus♂ × E. fuscoguttatus♀*). In addition, MDA is considered a lipid peroxidation marker used to determine lipid peroxidation as a result of increased oxidative stress [38,39]. Our study demonstrated that dietary cobalt levels (0.277 mg/kg–0.316 mg/kg) could significantly reduce hepatic MDA levels and could help to reduce the potential for oxidative stress, similar to the findings of Liu et al. [37], who showed that moderate amounts of cobalt could reduce liver MDA levels. This study thus revealed that dietary cobalt supplementation improved the antioxidant capacity of largemouth bass.

The Nrf2-Keap1 signaling pathway is one of the most classical pathways regulating the antioxidant system in animals, which can be induced by external oxidative stress conditions to maintain intracellular redox homeostasis and reduce cellular sensitivity to death signals [40]. In this study, although *nrf2* and *keap1* did not change significantly among the treatment groups, their downstream antioxidant factors (*sod* and *gpx*) were both modulated by dietary cobalt levels. In its normal state, Nrf2 is present in the cytoplasm and is maintained in a relatively stable state by the ubiquitination system [41], which, as explained here, in the absence of stimulation by external or internal conditions, may not cause changes in *nrf2* and *keap1*. However, *sod* and *gpx* are not only regulated by the Nrf2 signaling pathway but are also present in several signaling pathways (PI3K-Akt, MAPK, JAK-STAT, etc.) [42,43,44], which allows them to undergo an important antioxidative stress effect. In this study, the *sod* mRNA levels were significantly upregulated in the 0.201, 0.233, and 0.277 mg/kg dietary cobalt levels, and when the cobalt level reached 0.233 mg/kg, the *gpx* mRNA expression levels were upregulated. In mineral studies, dietary cobalt added to feeds has also been found to activate the antioxidant systems in organisms [45]. The expression levels of downstream genes *sod* and *gpx* were elevated in common carp (*Cyprinus carpio*) when Doum Palm Fruit Powder (DPFP) containing cobalt was added at 200 mg/kg [46]. Therefore, the addition of cobalt to the feed at a certain level can also improve antioxidant capacity by promoting the expression of antioxidant-related genes.

NF-κB has multiple biological functions and assists in complex biological processes; there are multiple mechanisms of action of NF-κB in the stress response, and it is involved in the inflammatory response and the development of stress-related diseases [47]. It has been demonstrated that mineral supplementation can activate the NF-κB signaling pathway [48,49]. In this study, the levels of some pro-inflammatory factors (*nfκb*, *tnfα*, *il-8*) did not change significantly, while anti-inflammatory factors (*tgf-β* and *il-10*) appeared to be upregulated. The NF-κB family of transcription factors is essential for the regulation of systemic immune pro-inflammatory responses, and the stimuli that initiate NF-κB activation are diverse but are usually attributed to pro-inflammatory cytokines and chemokines [50]. The non-significant changes in pro-inflammatory factors (*tnfα* and *il-8*) in this experiment might have led to the non-significant differences in *nf-κb*. Conversely, *tgf-β*, which is responsible for controlling cell proliferation and differentiation and the healing of wounds, is part of the immune system [51], and the highest expression level was observed in the 0.233 mg/kg dietary cobalt level, while *il-10* mRNA, which increased at the 0.201 mg/kg dietary cobalt level, is encoded by the IL-10 gene located on chromosome 1, and its expression implies its role in guarding the host against pathogens due to its anti-inflammatory properties [52,53]. The activity of these genes shows how dietary cobalt levels contributed to the improvement in the immune status of largemouth bass.

## 5. Conclusions

This study’s results present an improvement in the growth performance and feed utilization of largemouth bass fed with optimal dietary cobalt levels. Furthermore, cobalt has an important function in improving the antioxidant status and immune response of largemouth bass by regulating the Nrf2-Keap1 and NF-κB signaling pathways. Quadratic regression analyses based on the SGR and FCR showed that the optimal requirement was 0.24 and 0.26 mg/kg of dietary cobalt, respectively.

## Figures and Tables

**Table 1:** Experimental basic formula (%, dry matter).

Ingredients	Level (%)	Ingredients	Level (%)
Fish meal ^1^	20	Choline chloride	0.5
Casein ^1^	28	Vitamin premix ^2^	1
Gelatin ^1^	7	Mineral premix ^3^ (without cobalt)	1
Wheat flour ^1^	16	Calcium phosphate	4
Fish oil	4	Microcrystalline cellulose	14.45
Soybean oil	4	Vitamin C	0.05
Component analysis			
Crude protein (%)	46.38 ± 0.24		
Crude lipid (%)	9.86 ± 0.19		

^1^ The ingredients were obtained from Wuxi Tongwei Feedstuffs Co., Ltd., Wuxi, China. ^2^ Vitamin premix (IU or mg/kg of premix, purchased from HANOVE Biotechnology Co., LTD, Wuxi, China). ^3^ Mineral premix (without cobalt) (mg/kg of premix, purchased from HANOVE Biotechnology Co., LTD, Wuxi, China).

**Table 2:** Primer sequences for real-time PCR analysis in this work.

Genes		Primer Sequence (5′-3′)	Reference
*gapdh*	Forward	ACTGTCACTCCTCCATCTT	AZA04761.1
	Reverse	CACGGTTGCTGTATCCAA	
*tgf-β*	Forward	GCTCAAAGAGAGCGAGGATG	[22]
	Reverse	TCCTCTACCATTCGCAATCC	
*il-8*	Forward	CGTTGAACAGACTGGGAGAGATG	[23]
	Reverse	AGTGGGATGGCTTCATTATCTTGT	
*il-10*	Forward	CGGCACAGAAATCCCAGAGC	[23]
	Reverse	CAGCAGGCTCACAAAATAAACATCT	
*nrf2*	Forward	AGAGACATTCGCCGTAGA	NM_212855.2
	Reverse	TCGCAGTAGAGCAATCCT	
*keap1*	Forward	CGTACGTCCAGGCCTTACTC	XP_018520553.1
	Reverse	TGACGGAAATAACCCCCTGC	
*tnf-α*	Forward	CTTCGTCTACAGCCAGGCATCG	[22]
	Reverse	TTTGGCACACCGACCTCACC	
*sod*	Forward	TGGCAAGAACAAGAACCACA	[22]
	Reverse	CCTCTGATTTCTCCTGTCACC	
*gpx*	Forward	GAAGGTGGATGTGAATGGA	MK614713.1
	Reverse	CCAACCAGGAACTTCTCAA	
*nf-κb*	Forward	CCACTCAGGTGTTGGAGCTT	XP_027136364.1
	Reverse	TCCAGAGCACGACACACTTC	

**Table 3:** Growth performance and feed utilization.

Dietary Cobalt Levels (mg/kg)	IBW (g)	FBW (g)	WGR (%)	SGR (%/day)	FCR	SR (%)
0.129	1.68 ± 0.02	18.69 ± 0.10 ^a^	1013.58 ± 15.84 ^a^	4.30 ± 0.03 ^a^	1.08 ± 0.03 ^b^	91.67 ± 8.33
0.192	1.67 ± 0.01	20.24 ± 0.40 ^b^	1110.10 ± 30.06 ^b^	4.45 ± 0.04 ^b^	0.99 ± 0.01 ^a^	91.67 ± 3.33
0.201	1.69 ± 0.01	20.40 ± 0.10 ^b^	1111.91 ± 10.74 ^b^	4.45 ± 0.01 ^b^	0.99 ± 0.02 ^a^	95.00 ± 2.89
0.233	1.70 ± 0.01	20.58 ± 0.44 ^b^	1115.42 ± 28.72 ^b^	4.46 ± 0.04 ^b^	0.97 ± 0.01 ^a^	95.00 ± 2.89
0.277	1.67 ± 0.01	19.93 ± 0.24 ^b^	1097.37 ± 19.31 ^b^	4.43 ± 0.03 ^b^	0.99 ± 0.01 ^a^	93.33 ± 4.41
0.316	1.67 ± 0.01	19.71 ± 0.12 ^b^	1081.58 ± 11.72 ^b^	4.41 ± 0.07 ^b^	0.99 ± 0.01 ^a^	91.67 ± 1.67
F-value	0.200	0.061	0.217	0.233	0.352	0.622
*p*-value	0.467	0.005	0.037	0.022	0.007	0.981

Note: F-value, Homogeneity of Variance Test. Results are shown as mean values and standard error (±SE) (n = 3), significant differences between the six treatments are denoted by different letters (*p* < 0.05). IBW: initial body weight; FBW: final body weight; FCR = dry feed fed (g)/(FBW (g)-IBW (g)); WGR (%) = 100 × (FBW(g)-IBW(g))/IBW (g); SGR (% day^−1^) = 100 × [(Ln (FBW(g))-Ln (IBW (g)))/days]; SR (%) = 100 × (survival fish number/total fish number).

**Table 4:** Whole-body composition of largemouth bass fed with different levels of dietary cobalt.

Dietary Cobalt Levels (mg/kg)	Ash (%)	Lipid (%)	Moisture (%)	Protein (%)
0.129	3.40 ± 0.11	4.35 ± 0.89	72.99 ± 0.81 ^b^	14.92 ± 0.62 ^a^
0.192	3.63 ± 0.24	6.98 ± 0.59	71.36 ± 0.65 ^a^	18.70 ± 0.83 ^b^
0.201	3.55 ± 0.12	7.33 ± 0.55	71.50 ± 0.22 ^ab^	16.84 ± 1.35 ^ab^
0.233	3.52 ± 0.08	5.38 ± 1.58	71.88 ± 0.28 ^ab^	16.89 ± 1.84 ^ab^
0.277	3.69 ± 0.11	6.39 ± 0.17	71.51 ± 0.93 ^ab^	17.41 ± 0.27 ^ab^
0.316	3.35 ± 0.05	5.46 ± 1.41	71.89 ± 0.22 ^ab^	18.24 ± 0.71 ^ab^
F-value	0.404	0.483	0.284	0.097
*p*-value	0.579	0.240	0.048	0.045

Note: F-value, Homogeneity of Variance Test. Results are shown as mean values and standard error (±SE) (*n* = 3), significant differences between the six treatments are denoted by different letters (*p* < 0.05).

**Table 5:** Antioxidant enzymatic parameters of largemouth bass fed with different levels of dietary cobalt.

Dietary Cobalt Levels (mg/kg)	CAT (U/mgprot)	SOD (U/mgprot)	T-AOC (mmol/mgprot)	GSH (μmol/gprot)	GSH-Px (U/mgprot)	MDA (nmol/mgprot)
0.129	5.25 ± 0.65 ^ab^	5.91 ± 1.61	0.24 ± 0.06 ^a^	12.45 ± 1.51	6.05 ± 3.77 ^a^	0.77 ± 0.18 ^ab^
0.192	4.44 ± 0.51 ^ab^	3.35 ± 0.92	0.46 ± 0.03 ^b^	14.47 ± 1.04	5.98 ± 2.78 ^a^	0.79 ± 0.20 ^ab^
0.201	5.14 ± 0.71 ^ab^	6.33 ± 1.45	0.12 ± 0.04 ^a^	12.81 ± 2.75	5.21 ± 2.26 ^a^	0.95 ± 0.32 ^b^
0.233	6.24 ± 1.03 ^bc^	5.81 ± 1.59	0.12 ± 0.04 ^a^	15.23 ± 2.85	21.42 ± 4.41 ^b^	0.75 ± 0.16 ^ab^
0.277	8.21 ± 0.74 ^c^	6.98 ± 1.18	0.12 ± 0.04 ^a^	19.55 ± 3.07	15.06 ± 6.96 ^ab^	0.30 ± 0.10 ^a^
0.316	3.48 ± 0.61 ^a^	7.38 ± 1.28	0.20 ± 0.02 ^a^	14.62 ± 1.79	10.49 ± 4.72 ^ab^	0.33 ± 0.06 ^a^
F-value	0.838	0.704	0.368	0.311	0.185	0.077
*p*-value	0.001	0.490	0.000	0.267	0.028	0.048

Note: F-value, Homogeneity of Variance Test. Results are shown as mean values and standard error (±SE) (*n* = 9), significant differences between the six treatments are denoted by different letters (*p* < 0.05).

## Data Availability

The authors confirm that the data supporting the findings of this study are available within the manuscript, tables, and figures.
